# Knowledge of Salt, Oil, and Sugar Reduction (“Three Reductions”) and Its Association with Nutrition-Related Chronic Diseases in Chinese Adults: A Nationwide Cross-Sectional Study

**DOI:** 10.3390/nu17172766

**Published:** 2025-08-26

**Authors:** Yujie Qiu, Caicui Ding, Fan Yuan, Weiyan Gong, Tanchun Yu, Yan Zhang, Ailing Liu

**Affiliations:** National Institute for Nutrition and Health, Chinese Center for Disease Control and Prevention, Beijing 100050, China; qiuyj0323@163.com (Y.Q.); dingcc@ninh.chinacdc.cn (C.D.); yuanfan@ninh.chinacdc.cn (F.Y.); gongwy@ninh.chinacdc.cn (W.G.); yutc@ninh.chinacdc.cn (T.Y.); zhangyan@ninh.chinacdc.cn (Y.Z.)

**Keywords:** knowledge, adults, chronic disease, influencing factors, logistic models, cross-sectional study

## Abstract

**Background/Objectives**: Excessive intake of salt, oil, and sugar constitutes a major dietary risk factor for chronic diseases in China. Although salt, oil, and sugar reduction (“Three Reductions”) has been promoted at the national level, the population’s knowledge remains inadequately evaluated. This study aimed to assess the “Three Reductions” knowledge level among Chinese adults and its association with nutrition-related chronic diseases. **Methods**: Data were obtained from the Chinese Nutrition and Health Knowledge Survey 2022, a nationally representative cross-sectional study. A total of 68,673 participants aged 18–64 years were recruited from 200 survey sites of 31 provinces by multi-stage stratified random cluster sampling methods. A standard questionnaire was used for data collection, and multivariable logistic regression models were employed to examine factors associated with “Three Reductions” knowledge and its relationship with chronic diseases. All analyses were weighted by complex sampling. **Results**: In 2022, the mean “Three Reductions” knowledge score was 16.43 ± 4.17 (the full score is 24 points), and the awareness rate was 49.3% (95% CI: 47.0–51.6%). Females, those with higher education and income levels, those employed in medical institutions, and residents in urban and eastern areas had higher “Three Reductions” levels (*p* < 0.05). The “Three Reductions” knowledge level was significantly associated with chronic disease risk (*p* < 0.05). Specifically, the risk of chronic disease was reduced by 5% in the medium-score group (OR = 0.95, 95% CI: 0.90–1.00) and by 11% in the high-score group (OR = 0.89, 95% CI: 0.84–0.95). **Conclusions**: “Three Reductions” knowledge among Chinese is at a moderate level, with a significant association between “Three Reductions” knowledge level and chronic disease status. Dissemination of “Three Reductions” knowledge, especially practical knowledge, should be enhanced at the national level through various routes to reduce chronic disease risk.

## 1. Introduction

Non-communicable diseases (NCDs) represent the leading global cause of mortality and disability, accounting for over half of the global burden of disease [1,2]. In China, 10.6 million deaths and 349.3 million disability-adjusted life-years (DALYs) are due to NCDs, accounting for 91.0% of all deaths and 86.7% of all DALYs [3]. The crude incidence rate of cardiovascular disease (CVD) among Chinese adults was 620.33 per 100,000, while the crude prevalence rates of hypertension and diabetes were 27.9% and 11.2%, respectively [4,5]. Among modifiable risk factors for NCDs, suboptimal diet, characterized by high salt, high oil, and high sugar intake, stands out as a critical and preventable factor. Excessive consumption of salt and oil is associated with CVD and type 2 Diabetes, contributing to 51.0% of CVD and type 2 diabetes mortality cases among Chinese adults [6,7,8,9]. There was a near doubling of deaths attributed to excessive sugar-sweetened beverage (SSB) intake among Chinese residents from 1990 to 2019 [10]. In 2015, Chinese adults consumed 9.3 g/day of cooking salt and 43.2 g/day of cooking oil. Carbohydrates and fat contribute 53.4% and 34.6% of total daily energy intake, respectively [11,12]. While slight declines were observed by 2022, with salt intake declining to 8.3 g/day and cooking oil to 41.1 g/day [13], these consumption levels still substantially exceed the dietary recommendations of <5 g/day for salt and 25–30 g/day for oil [14]. Although the intake of added sugar is not high, the consumption of SSBs is constantly increasing [15]. Substantial evidence confirms that reducing salt, oil, and added sugar intakes can significantly lower risks of CVDs, type 2 diabetes, and obesity [16,17,18,19]. Notably, reducing daily salt intake in China by 1 g could prevent almost 9 million cardiovascular events by 2030 [20]. The necessity for reduction in the Chinese context is underscored by the significant gap between current consumption and guidelines.

Knowledge, especially nutrition knowledge (NK), plays an important role in changing the pathway to suboptimal diets and health. Several studies have reported a significant, positive association between NK and dietary intake or pattern in adults [21,22,23]. Studies with larger samples and validated instruments used to measure NK or dietary intake more often observed significant positive associations [24]. In addition, for health outcomes, some studies found that NK was one of the important determinants of overweight and obesity, which was significantly associated with a lower prevalence of obesity [22,25]. In particular, research has shown that salt-related knowledge is negatively correlated with 24-h urinary sodium excretion, indicating that increasing knowledge can be an effective approach to reducing salt intake [26]. Therefore, widespread education and knowledge dissemination is a promising strategy for reducing consumption of cooking oil, cooking salt, and added sugar, and thereby preventing chronic disease.

Given the importance of NK, especially regarding the reduction in salt, oil, and sugar, the Chinese government launched the “China Healthy Lifestyle for All” initiative in 2016. This campaign put forward the concept of “reduced salt, reduced oil, reduced sugar” (“Three Reductions”) and stressed the importance of a reasonable diet in the prevention of chronic diseases, to guide the public to enhance public knowledge of “Three Reductions” and to form healthier eating habits through the popularization of the “Three Reductions” [27]. The “Three Reductions” initiative has been widely publicized and implemented in China across multiple public health promotion measures, including social media disseminations, community health education programs, and the establishment of supportive environments such as nutrition-focused canteens. Nevertheless, evidence regarding the campaign’s effectiveness in improving public knowledge of the “Three Reductions” remains limited. Moreover, few studies have examined the association between knowledge and the risks of chronic diseases in China.

Therefore, the objective of this study is to evaluate the “Three Reductions” knowledge level and its association with chronic disease status based on the nationally representative data from the China Nutrition and Health Knowledge Survey (CNHKS). The findings will provide empirical evidence to guide targeted nutrition education interventions and support the refinement of chronic disease prevention and management strategies in China.

## 2. Materials and Methods

### 2.1. Data Source

The data in this study were obtained from the China Nutrition and Health Knowledge Survey (CNHKS) among Chinese adults in 2022, which was a nationally representative survey of nutrition knowledge (NK) among general Chinese adults aged 18 to 64. The survey was conducted by using a multi-stage stratified random cluster sampling method to select residents aged 18 to 64 from 200 survey sites from 31 provinces (municipalities or autonomous regions) in China. Within each survey site, 3 townships were randomly chosen, followed by the random selection of 2 villages or residential communities per township. From each village, 70 households were randomly sampled, and one resident aged 18–64 years was selected from each household. Trained interviewers conducted the face-to-face interviews in the participants’ homes. Participants were eligible if they: (1) were aged 18–64 years; (2) had lived at their current residence for ≥6 months of the year; and (3) were able to communicate normally without serious intellectual disabilities. The inclusion criteria for this specific analysis were: completed data on all survey components, including basic information and NK. Exclusion criteria included participants with missing demographic variables (gender, age, etc.) and missing “Three reduction” scores, scores exceeding the maximum possible value, or those who reported “don’t know” for chronic disease status. Finally, a total of 68,673 participants were included in this study. The study was conducted in accordance with the Declaration of Helsinki and approved by the Medical Ethical Review Committee at the National Institute for Nutrition and Health, Chinese Center for Disease Control and Prevention (No. 2022-037). All respondents agreed to participate in this survey after fully understanding the content and meaning of the survey.

### 2.2. Data Collection

“Three Reductions” knowledge was measured using a validated questionnaire named the Chinese Nutrition Health Knowledge Questionnaire for adults (CNHKQ), a standardized questionnaire developed by the national project team specifically for the CNHKS. Its development followed a rigorous scientific process. Delphi expert consultation and the pilot test demonstrated good reliability (Cronbach’s α = 0.85, split-half r = 0.86) and validity [28,29]. The CNHKQ consisted of two parts. The first part included demographic information such as gender, birthdate, education level, occupation, marital status, income, height, weight, and chronic disease status (including hypertension, diabetes, dyslipidemia, coronary heart disease, stroke, and other chronic diseases related to nutrition). Chronic patients were those who self-reported having one or more chronic diseases. Weight status was determined by body mass index (BMI), which was calculated as weight in kilograms divided by height in meters squared. This study adopted the BMI classification criteria established by the Working Group on Obesity in China: underweight (BMI < 18.5 kg/m^2^), normal weight (18.5 kg/m^2^ ≤ BMI < 24.0 kg/m^2^), overweight (24.0 kg/m^2^ ≤ BMI < 28.0 kg/m^2^), and obesity (BMI ≥ 28.0 kg/m^2^) [30]. The second part contained 20 items with 65 options about NK. Among them, the twelve options related to salt, oil, and added sugar were selected to form the “Three Reductions” knowledge score. The specific contents cover the recommended intake levels of cooking salt, cooking oil, and added sugar, as well as the disease risk of excessive intake of salt, oil, and sugar. We used these items to assess the residents’ “Three Reductions” knowledge level. The full content of the items is detailed in the Section 3.3. Out of a maximum score of 24, “2” points were given for a correct answer, and “0” points for the other answers. Correct responses from each option were added to give an overall score. Participants were considered as having “Three Reductions” knowledge when the score was higher than 75 percent of the full score (namely, 18 points and over) and vice versa [29]. The “Three Reductions” awareness rate was defined as the proportion of participants who had knowledge among the total participants. A higher score and awareness rate reflected a higher “Three Reductions” knowledge level.

In addition, the provincial variables of urbanization rate, GDP, and per capita health expenditure were collected from the China Statistical Yearbook 2023 [31] and China Health Care Statistics Yearbook 2022 [32].

### 2.3. Statistical Analysis

All statistical analyses were conducted using SAS software (version 9.4, SAS Institute Inc., Cary, NC, USA), incorporating adjustments for the complex sampling weight, stratification, and clusters. Descriptive analysis was conducted using PROC SURVEYMEANS and PROC SURVEYFREQ. The “Three Reductions” knowledge score was described using the mean and standard deviation (SD). The weighted “Three Reductions” awareness rate was presented as a percentage with a 95% confidence interval (95% CI). The analysis of the variability of scores among subgroups was performed by an independent sample t-test and analysis of variance (ANOVA). Post hoc pairwise comparisons of scores were conducted using Tukey’s Honestly Significant Difference (HSD) test. The Rao–Scott chi-square test was used to compare the “Three Reductions” awareness rates between or among the subgroups. Post hoc pairwise comparisons were performed using the Bonferroni correction to adjust for multiple comparisons.

Multivariable logistic regression was applied to examine the potential factors of the “Three Reductions” knowledge awareness rate (awareness = 1, unawareness = 0). Independent variables included gender; age (18–29, 30–39, 40–49, 50–59, 60–64 years) [33]; education level (primary school or below [≤6 years of schooling]; junior high school [6–9 years of schooling], senior high school [9–12 years of schooling], junior college [14–15 years of schooling], and bachelor degree or above [≥16 years of schooling]); occupation (medical institutions, food industries, education institutions, and others); marital status (unmarried, married, and divorced or widowed); income level; weight status (underweight, normal, overweight, obesity); rural or urban residential area; and region.

Two-level logistic regression was then used to calculate the odd ratios (ORs) and 95% Cl for the association between the “Three Reductions” knowledge level and chronic disease status (no = 0, yes = 1). Three models were used to estimate ORs in this study. Model 1 was crude without any adjustment. Model 2 adjusted for demographic variables (gender, age, education, marital status, income, weight status, and occupation), and model 3 was further adjusted for provincial-level variables (urban or rural, region, urbanization rate, GDP, and per capita health expenditure). In addition, we carried out stratified analyses by gender and age to further conducted to explore potential effect modifiers. Knowledge level, income level, urbanization rate, GDP, and per capita health expenditure are categorized as tertiles (low: ≤*P*_33.3_, medium: *P*_33.3_–*P*_66.7_, high: ≥*P*_66.7_). A *p*-value < 0.05 was considered statistically significant.

## 3. Results

### 3.1. Basic Characteristics

The basic characteristics of the participants are shown in Table 1. A total of 68,673 participants were included in this study, and 34,927 (48.8%) participants were females. The largest number was in the 50–59 age group (24.4%). A total of 34,074 (67.0%) participants were from urban areas, while 25,226 (36.7%) lived in eastern China, 40.9% were overweight or obesity, and 6109 (11.0%) were employed in medical institutions. More than 55% of participants had a senior school education or higher.

### 3.2. “Three Reductions” Knowledge Level

As shown in Table 1, the mean score of the participants was 16.43 ± 4.17. Females scored significantly higher than males, at 16.56 ± 4.19 and 16.31 ± 4.14, respectively (*p* < 0.001). Scores increased with the education level and income level (*P*_trend_ < 0.001). Specifically, the scores of the high-income group (16.91 ± 4.00) were significantly higher than those of the medium-income group (16.32 ± 4.29) and low-income group (15.89 ± 4.18) (*p* < 0.001). However, there was no statistically significant difference in the scores between the low and medium income groups (*p* = 0.177). With regard to the occupation, those employed in medical institutions had the highest mean score at 17.30 ± 4.02, followed by those in education (score 16.53 ± 4.46). Additionally, significant differences were observed in scores among participants who lived in different areas, with higher scores among participants in the urban area (score 16.75 ± 4.03) or eastern region (score 16.91 ± 4.01) (*p* < 0.01). The comparison of scores across weight status and marital status revealed no statistically significant differences (*p* > 0.05).

The “Three Reductions” awareness rate was 49.3% (95% CI: 47.0–51.6%). Females had a greater rate (50.8%, 95% CI: 48.5–53.1%) than males (47.8%, 95% CI: 45.4–50.3%). The rate increased with the education level and income level (*P*_trend_ < 0.001). Regarding the occupation, participants who were employed in medical institutions had the highest rate (58.9%, 95% CI: 55.9–62.2%). Additionally, the rate varied by area, with participants in the eastern region (53.1%, 95% CI: 50.1–56.1%) and urban areas (52.2%, 95% CI: 49.3–55.1%) (*p* < 0.001) showing higher rates.

**Figure 1:**
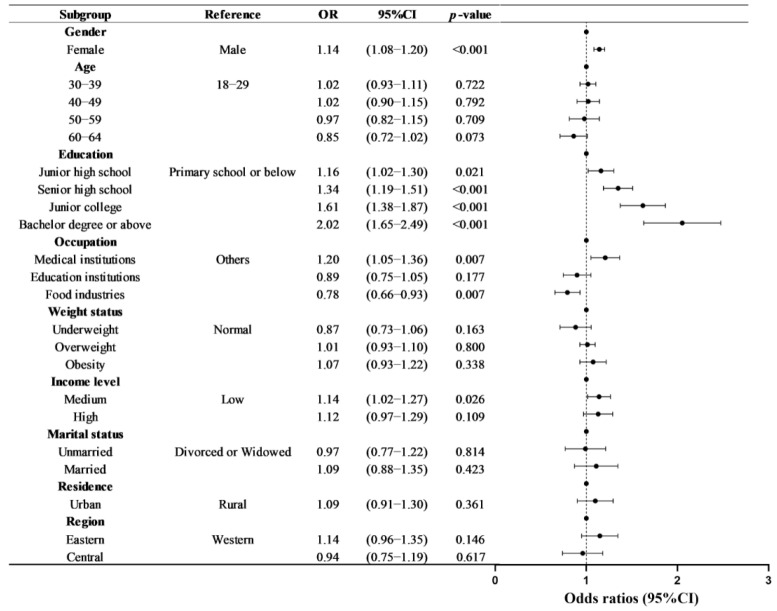
Factors associated with “Three Reductions” knowledge level: estimated from binary logistic regression.

In multivariable analysis, the odds of having “Three Reductions” knowledge for females were 14% higher than for males (OR = 1.14, 95% CI: 1.08–1.20). The “Three Reductions” awareness rate generally increased with higher education level (*P*_trend_ < 0.001). Participants who were employed in medical institutions (OR = 1.20, 95% CI: 1.05–1.36) had higher rates than others. Compared to the low-income level group, people with a medium income level have a higher awareness rate (OR = 1.14, 95% CI: 1.02–1.27) (Figure 1).

### 3.3. The “Three Reductions” Knowledge Items

The accurate rates of the 12 items on the “Three Reductions” knowledge are listed in Table 2 from highest to lowest. The highest accuracy rate was the item “There is no harm in eating more oil, salt and added sugar” (90.51%), followed by the item “The association of excessive salt intake with the risk of hypertension.” (87.08%). The accurate rate of excessive oil, salt, and added sugar intakes increases disease risk was more than 60%. The lowest accurate rate was the title “The recommended daily intake of cooking oil.” (31.40%). The accurate rates of the recommended intake of salt and added sugar were 58.81% and 62.02%, respectively (Table 2).

### 3.4. The Association with “Three Reductions” Knowledge Level and Nutrition-Related Chronic Diseases

In the multivariate adjusted model, the “Three Reductions” knowledge level was significantly associated with the risk of chronic disease status. Specifically, the risk of chronic disease decreased by 5% in the medium-score group (OR = 0.95, 95% CI: 0.90–1.00, *p* = 0.049) and by 11% in the high-score group (OR = 0.89, 95% CI: 0.84–0.95, *p* < 0.001) (Table 3). No association was found between each specific disease and the “Three Reductions” knowledge level.

Stratified analyses demonstrated a significant inverse association between “Three Reductions” knowledge level and chronic disease status among females (Q2 vs. Q1: OR = 0.91, 95% CI: 0.84–0.98, *p* = 0.018; Q3 vs. Q1: OR = 0.86, 95% CI: 0.79–0.94, *p* = 0.002). No significant association was observed among males. Age-specific analysis showed progressively attenuated effects with advancing age: a strong inverse association in participants aged 18–39 years (Q2 vs. Q1: OR = 0.78, 95% CI: 0.68–0.87, *p* < 0.001; Q3 vs. Q1: OR = 0.74, 95% CI: 0.64–0.84, *p* < 0.001), a marginal association in those aged 40–59 years (Q2 vs. Q1: OR = 0.95, 95% CI: 0.89–1.02, *p* = 0.174; Q3 vs. Q1: OR = 0.88, 95% CI: 0.81–0.95, *p* = 0.002), and no significant association in those aged 60–64 years (Table 4).

## 4. Discussion

Our study found that the mean “Three Reductions” knowledge score was 16.43, and the awareness rate was 49.3%. The public’s awareness of “Three Reductions” knowledge was at a moderate level. This demonstrated that “Three Reductions” knowledge has been well disseminated since the implementation of “China Healthy Lifestyle for All” in 2016. In addition, with the rapid development of the Chinese economy and the improvement of living standards, people pay more attention to their health management and have a greater concern for healthy eating [34,35]. However, there is still much room for enhancing the people’s knowledge level. At the national level, diversifying knowledge dissemination channels, enriching content, and developing innovative formats are all imperative.

Our study demonstrated that the knowledge level varied by demographic. In particular, gender and education level are important factors affecting knowledge level, which has already been observed in several earlier studies [29,35,36,37,38]. For one thing, this may be because females take body management and weight control more seriously. On the other hand, females mostly take responsibility for preparing meals at home, including food purchasing and cooking [39,40]. Therefore, they will pay more attention to related knowledge, such as reducing salt, oil, and sugar. The important role of education levels could be attributed to the fact that education is an important determinant of an individual’s ability to understand and respond to a wide range of health information [29]. These demographic differences illustrate that nutrition education should focus on people who are male, older, have lower education levels or income levels, non-single status, and live in rural or western regions. Notably, however, the relationship between age and nutrition knowledge (NK) was contradictory across studies [41,42,43]. Our study did not identify a significant association between age and knowledge level. This might be related to the diversification of information channels and the comprehensiveness of knowledge popularization [44]. Various formats such as television, community publicity, new media, and the Internet have made it easier for people to acquire knowledge. People of all ages can access information about “Three reductions” through these channels, regardless of their age. In addition, health education has been widely popularized, covering places such as schools [45], communities [46], and medical institutions. Those allow people of all age groups to obtain relevant knowledge, which has, to a certain extent, weakened the influence of age on the knowledge level.

We also showed that the participants’ accurate rates varied on different items. Firstly, it appeared that the key dietary guidelines recommendations were well known to the general public, such as eating less processed meat products (63.84%). However, less than a third of the population was aware of the recommended intake of cooking oil (31.40%). This is similar to perceptions in another study [47]. Different people often have different definitions of “eat less” and “eat more”, and specific and quantified numbers can better guide eating practice. This finding reflects dissemination of some quantitative food knowledge is necessary and needs to be strengthened. Secondly, our study found that the awareness of the recommended salt intake was 58.81%, which was a significant improvement over 2015 (6.1%) [34]. However, the cooking salt intake of Chinese adults (8.3 g/d) was still excessive, nearly twice the recommended intake (5 g/d) [13]. This ‘know-to-do gap’ phenomenon may be closely related to the type of knowledge [38,41]. Cognitive psychology divides knowledge into two types. These are: (1) declarative knowledge, also known as knowledge of “know-what”. For example, consumers need to know the recommended salt intake and the disease risks of excessive salt intake; and (2) procedural knowledge, or “know-how” knowledge. For example, how to select low-salt foods by reading food labels and how to reduce their salt intake in home cooking by using spices or salt-control spoons [48,49]. Several studies concluded that procedural NK is more closely associated with changes in dietary behavior than declarative NK [50]. Declarative NK may have no relationship to choosing a healthy diet [24]. The Chinese often knew better in declarative knowledge and lacked practical guidelines; they could not effectively combine dietary information with their dietary and exercise behavior [51]. Hendrie et al. similarly reported that the knowledge of how to reduce fat intake was good, but knowledge of the energy density of fat, the type of fat to cut down on, and types of foods low or high in fat was poor [47]. These findings suggest that if the dissemination of “Three Reductions” knowledge focuses purely on knowledge of facts (declarative knowledge) rather than procedural knowledge, it may be less effective in eliciting positive dietary change. Lastly, our research revealed that people were generally able to perceive the severity of chronic diseases caused by excessive intake of oil, salt, and added sugar. The percentage of accurate rates on all relevant items exceeded 60%. However, according to the Health Belief Model, perceiving severity is not enough; it is also important to perceive susceptibility, benefits, and barriers, and have the confidence to overcome them (self-efficacy) to truly change dietary behaviors [52,53,54]. Therefore, nutrition education should not only disseminate practical knowledge but should be oriented towards changing dietary behaviors by increasing the audience’s self-efficacy in various ways and building practical skills, including using salt restriction spoons and reading nutrition labels to identify low-sodium, low-fat, and low-sugar food products to guide long-term behavioral changes [55]. Meanwhile, improving residents’ dietary habits requires a multi-pronged approach. Besides individual nutrition education, enhancing access to nutritious foods (especially in underdeveloped areas) and strengthening food marketing regulations are also essential to support such changes [56].

Our study found a significant association between the “Three Reductions” knowledge level and chronic disease status, which was consistent with other related studies. One previous study conducted in Wuhan found a significant negative correlation between nutrition knowledge and diabetes/hyperglycemia, hypertension, coronary heart disease, and stroke (all *p* < 0.001) [57]. Similarly, a study from Spain found that increased NK was associated with healthier eating habits and decreased cardiovascular risk [58]. However, as a cross-sectional study, we acknowledge that these observed associations cannot establish causality or temporal relationships. The negative correlation might reflect that individuals with chronic diseases become more health-conscious [59,60]. Furthermore, it is undeniable that the associations were generally weak. This may be due to chronic diseases are influenced by a combination of risk factors, including behavioral, environmental, occupational, and metabolic factors [61]. This was confirmed by a stratified analysis of gender and age. Females had a significant negative association, while males did not. This could be because, compared to females, males are more likely to have unhealthy habits such as smoking and drinking, which can weaken the protective effect of knowledge against chronic diseases. With age, physiological changes such as declined organ functions and metabolic rates, the accumulation of underlying diseases, and long-standing unhealthy lifestyle habits play a stronger role in chronic diseases, diminishing the protective effect of knowledge. These findings stress the need for targeted health education based on demographics. Nevertheless, knowledge should be given more importance as a key modifiable risk factor than the immutable factors such as age, gender, and education.

To our knowledge, this is the first study to analyze the “Three Reductions” knowledge level among the nationally representative Chinese population. The large sample size was a strength as it enabled the analysis to detect correlations adequately and more representatively. Likewise, provincial-level variables were also collected so that areas of weak knowledge could be identified. The study, however, is not without limitations. First, as a cross-sectional study, the findings should be interpreted as correlations rather than establishing causality or temporal relationships. Consequently, while this design is highly suitable for assessing the knowledge level, it is not appropriate for formal risk assessment of chronic disease status, which would require longitudinal or nested case-control designs. Second, it is important to acknowledge that some risk factors for chronic diseases, including eating, smoking, and drinking, were not collected. The independent variables in all models still do not explain most of the variation in the dependent variable. Lastly, the use of secondary data in this study may cause potential information bias, particularly as the chronic disease status was self-reported, and reporting bias might have occurred.

## 5. Conclusions

Our research results show that knowledge of the “Three Reductions” among the Chinese population is moderate, with lower levels found in males, older people, those with lower education or income, non-single status, and residents of western or rural areas. “Three Reductions” knowledge is significantly associated with chronic disease status. To improve awareness of the “Three Reductions” to prevent and control chronic disease, targeted strategies for knowledge dissemination are essential, such as using social media platforms, community workshops, and healthcare collaborations. Schools and workplaces can also play a key role in integrating “Three Reductions” education. Additionally, enhancing policy measures, including Front of Package (FOP) labeling systems that highlight salt, fat, and sugar content on packaged food, can raise consumer awareness and further support behavior change.

## Figures and Tables

**Table 1:** Univariate analysis of participants’ knowledge level on Salt, Oil, and Sugar Reduction (“Three Reductions”).

Characteristics	Samples, *n* (%) *	Score ^†^		Awareness Rate ^§^	
		Mean ± SD	*p*-Value	%(95% CI)	*p*-Value
Overall	68,673 (100.0)	16.43 ± 4.17		49.3 (47.0, 51.6)	
Gender			<0.001		<0.001
Male	33,746 (51.2)	16.31 ± 4.14		47.8 (45.4, 50.3)	
Female	34,927 (48.8)	16.56 ± 4.19		50.8 (48.5, 53.1)	
Age (years)			<0.001		<0.001
18–29	11,282 (21.2)	16.74 ± 4.07 ^ab^		52.0 (49.7, 54.3) ^ab^	
30–39	18,560 (24.0)	16.78 ± 3.99 ^a^		52.5 (50.2, 54.8) ^a^	
40–49	14,445 (22.3)	16.49 ± 4.17 ^b^		50.0 (47.2, 52.8) ^b^	
50–59	16,666 (24.4)	16.03 ± 4.27		45.7 (42.7, 48.7)	
60–64	7720(8.2)	15.63 ± 4.40		41.8 (38.4, 45.2)	
Education			<0.001		<0.001
Primary school or below	12,504 (14.0)	15.37 ± 4.39		39.6 (37.1, 42.2)	
Junior high school	24,012 (29.8)	15.91 ± 4.21		44.3 (41.7, 46.8)	
Senior high school	13,116 (20.8)	16.41 ± 4.12		48.8 (45.9, 51.8)	
Junior college	10,654 (18.4)	17.01 ± 3.97		54.4 (51.4, 57.5)	
Bachelor degree or above	8373 (17.0)	17.59 ± 3.76		61.0 (57.9, 64.0)	
*P*_trend_		<0.001		<0.001	
Occupation			<0.001		<0.001
Medical institutions	6109 (11.0)	17.30 ± 4.02		58.9 (55.6, 62.2)	
Education institutions	2372 (4.1)	16.53 ± 4.46 ^a^		52.0 (46.4, 57.7) ^a^	
Food industries	5229 (7.8)	15.65 ± 4.43		43.3 (38.3, 48.3) ^b^	
Others	54,963 (77.1)	16.38 ± 4.12 ^a^		48.4 (46.3, 50.4) ^ab^	
Marital			0.083		0.097
Unmarried	8532 (15.1)	16.61 ± 4.14		51.3 (48.1, 54.5)	
Married	56,838 (81.2)	16.42 ± 4.15		49.0 (46.7, 51.4)	
Divorced or Widowed	3263 (3.6)	15.74 ± 4.65		45.0 (38.7, 51.3)	
Weight status			0.714		0.573
Underweight	2839 (4.1)	16.49 ± 4.24		47.6 (43.1, 52.0)	
Normal	37,839 (55.1)	16.41 ± 4.16		49.6 (46.7, 52.5)	
Overweight	22,236 (32.4)	16.42 ± 4.17		48.7 (46.6, 50.7)	
Obesity	5736 (8.4)	16.57 ± 4.10		50.5 (47.7, 53.3)	
Income level			<0.001		<0.001
Low	24,053 (30.3)	15.89 ± 4.18 ^a^		43.8 (41.0, 46.7)	
Medium	22,228 (29.5)	16.32 ± 4.29 ^a^		49.0 (46.2, 51.7)	
High	22,392 (40.3)	16.91 ± 4.00		53.6 (50.4, 56.7)	
*P*_trend_		<0.001		<0.001	
Residence			<0.001		0.004
Urban	34,074 (67.0)	16.75 ± 4.03		52.2 (49.3, 55.1)	
Rural	34,599 (33.0)	15.77 ± 4.35		43.4 (40.5, 46.3)	
Region			0.007		0.023
Eastern	25,226 (36.7)	16.91 ± 4.01		53.1 (50.1, 56.1)	
Central	21,978 (32.0)	15.85 ± 4.44 ^a^		45.8 (40.2, 51.5) ^a^	
Western	21,469 (31.3)	16.33 ± 3.99 ^a^		47.3 (44.1, 50.5) ^a^	

* Percentages are weighted. ^†^ The overall score is 24. ^§^ “Three Reductions” awareness rate: The proportion of participants who have “Three Reductions” knowledge (the score is higher than 18 points) to total participants. ^a,b^ Post hoc pairwise comparisons were performed for variables with significant overall differences (*p* < 0.05). No significant subgroup differences (*p* > 0.05 after adjustment) are indicated by the same superscript letters.

**Table 2:** The content and accurate rates of the items about “Three Reductions” in the questionnaire (descending order).

Items	Full Score	Score (Mean ± SD)	Accurate Rate (%)
There is no harm in eating more oil, salt and added sugar.	2	1.81 ± 0.59	90.51
The association of excessive salt intake with the risk of hypertension.	2	1.74 ± 0.67	87.08
The association of excessive cooking oil intake with the risk of obesity.	2	1.74 ± 0.67	86.96
The association of drinking excessive sugary drinks with the risk of obesity and dental caries.	2	1.63 ± 0.78	81.36
Recommendation for the intake levels of foods or beverages with added sugar.	2	1.38 ± 0.93	69.00
The association of less processed meat products with the risk of cancer.	2	1.35 ± 0.94	67.70
The association of excessive animal fat with the risk of strokes.	2	1.28 ± 0.96	63.95
Recommendation for the intake levels of processed meat products.	2	1.28 ± 0.96	63.84
The recommended daily intake of added sugar.	2	1.24 ± 0.97	62.02
Foods that contain more cooking oil and salt.	2	1.18 ± 0.98	58.84
The recommended daily intake of salt.	2	1.18 ± 0.98	58.81
The recommended daily intake of cooking oil.	2	0.63 ± 0.93	31.40

**Table 3:** The association between “Three Reductions” knowledge level and chronic disease status.

	Model 1	Model 2	Model 3
“Three Reductions” knowledge level (ref: Low)			
Medium	0.84 (0.80, 0.88) **	0.96 (0.91, 1.01)	0.95 (0.90, 1.00) *
High	0.75 (0.70, 0.79) **	0.91 (0.85, 0.96) **	0.89 (0.84, 0.95) **
Gender (ref: male)			
Female		0.85 (0.81, 0.88) *	0.84 (0.81, 0.88) **
Age (years) (ref: 18–29)			
30–39		1.53 (1.35, 1.70) **	1.49 (1.32, 1.66) **
40–49		3.33 (2.96, 3.71) **	3.21(2.85, 3.57) **
50–59		8.24 (7.34, 9.16) *	7.79 (6.92, 8.65) **
60–64		12.17 (10.76, 13.59) **	11.41 (10.07, 12.74) **
Education (ref: Primary School or Below)			
Junior high school		0.84 (0.78, 0.88) *	0.81 (0.77, 0.86) **
Senior high school		0.92 (0.85, 0.98) *	0.84 (0.78, 0.90) **
Junior college		0.74 (0.68, 0.81) *	0.67 (0.61, 0.73) **
Bachelor degree or above		0.70 (0.63, 0.77) *	0.62 (0.55, 0.68) *
Occupation (ref: others)			
Food and restaurant industries		1.27 (1.09, 1.46) *	1.15 (0.96, 1.34)
Education institutions		0.93 (0.79, 0.94) *	0.91 (0.80, 1.01)
Medical and health institutions		0.87 (0.79, 0.94) *	0.87 (0.79, 0.94) **
Marital (ref: Unmarried)			
Married		1.34 (1.21, 1.48) *	1.36 (1.22, 1.50) **
Divorced or Widowed		2.26 (1.96, 2.56) *	2.26 (1.96, 2.56) **
Weight status (ref: Underweight)			
Normal		0.83 (0.73, 0.93) **	0.82 (0.72, 0.93) *
Overweight		1.32 (1.16, 1.49) *	1.31 (1.15, 1.48) **
Obesity		2.32 (2.00, 2.63) **	2.31 (2.00, 2.62) **
Income level (ref: Low)			
Medium		0.87 (0.83, 0.92) **	0.83 (0.79, 0.88) *
High		0.86 (0.81, 0.91) **	0.78 (0.73, 0.82) *
Residence (ref: rural)			
Urban			1.33 (1.26, 1.40) *
Region (ref: western)			
Central			0.31 (0.31, 0.32) *
Eastern			0.91 (0.90, 0.91) *
Urbanization rate (ref: low)			
Medium			0.77 (0.52, 1.01)
High			0.87 (0.55, 1.18)
GDP (ref: low)			
Medium			0.75 (0.54, 0.96) *
High			0.60 (0.41, 0.80) *
Per capita health expenditure (ref: low)			
Medium			1.42 (0.91, 1.92)
High			1.65 (1.08, 2.21) *

* *p* < 0.05, ** *p* < 0.001. Model 1: Two level logistic regression unadjusted covariates, random effects σ*_p_*^2^ = 0.148, SE = 0.02; Model 2: Two level logistic regression adjusted for gender, age, education, occupation, marital, weight status, and income level, random effects σ*_p_*^2^ = 0.148, SE = 0.04; Model 3: Two level logistic regression adjusted for gender, age, education, occupation, marital status, weight status, and income level, urban/rural, region, and province-level risk factors, random effects σ*_p_*^2^ = 0.08, SE = 0.02.

**Table 4:** Stratified analyses of the association between “Three Reductions” knowledge level and chronic disease status *.

	“Three Reductions” Knowledge Level (Ref: Low)	OR	95% CI	*p*-Value
Gender				
Male	Medium	0.99	0.92, 1.07	0.831
	High	0.94	0.86, 1.02	0.125
Female	Medium	0.91	0.84, 0.98	0.018
	High	0.86	0.79, 0.94	0.002
Age (years)				
18–39	Medium	0.78	0.68, 0.87	<0.001
	High	0.74	0.64, 0.84	<0.001
40–59	Medium	0.95	0.89, 1.02	0.174
	High	0.88	0.81, 0.95	0.002
60–64	Medium	1.07	0.94, 1.20	0.241
	High	1.09	0.94, 1.25	0.203

* Two-level logistic regression adjusted for gender, age, education, occupation, marital status, weight status, and income level, urban/rural, region, and province-level risk factors.

## Data Availability

The original contributions presented in the study are included in the article. Further inquiries can be directed to the corresponding author.
